# Cardiorenal Biomarkers and Cerebrovascular Risk in Patients with Congenital Heart Disease

**DOI:** 10.3390/jcm15062440

**Published:** 2026-03-23

**Authors:** Efrén Martínez-Quintana, Fayna Rodríguez-González

**Affiliations:** 1 Cardiology Service, Complejo Hospitalario Universitario Insular-Materno Infantil, Avd. Marítima del Sur s/n, 35016 Las Palmas de Gran Canaria, Spain; 2 Department of Medical and Surgical Sciences, Faculty of Health Sciences, Universidad de Las Palmas de Gran Canaria, 35016 Las Palmas de Gran Canaria, Spain; 3 Hospital Universitario de Gran Canaria Dr. Negrín, 35010 Las Palmas de Gran Canaria, Spain

**Keywords:** congenital heart disease, ischemic stroke, NT-pro-BNP, dyslipidemia, risk stratification

## Abstract

**Background/Objectives**: Adults with congenital heart disease (CHD) have a substantially higher risk of ischemic stroke than the general population. Circulating biomarkers such as N-terminal pro B-type natriuretic peptide (NT-pro-BNP), high-sensitivity C-reactive protein (hs-CRP), and microalbuminuria have been associated with adverse cardiovascular outcomes in CHD, but their role in predicting cerebrovascular events remains uncertain. **Methods**: Prospective cohort study including 372 adults with CHD [median age 34 years (IQR 23–42); 57.8% male] followed at a tertiary center between 2017 and 2022. Baseline assessments included demographic characteristics, CHD anatomical complexity, cardiovascular risk factors, NT-pro-BNP, hs-CRP, lipid profile, and 24-h urinary albumin excretion. The primary endpoint was incident ischemic stroke during a median follow-up of 6.3 years (IQR 3.9–8.3). Univariable Cox proportional hazards models were used to identify predictors of stroke. **Results**: During follow-up, 13 patients (3.5%) experienced ischemic stroke. Patients with stroke were significantly older [51 (46–64) vs. 30 (23–40) years; *p* < 0.001] and had a higher prevalence of dyslipidemia (61.5% vs. 15.0%; *p* < 0.001). NT-pro-BNP levels were markedly higher in patients with stroke [369 (218–604) vs. 64 (21–172) pg/mL; *p* < 0.001]. No significant differences were observed between groups in renal function parameters, hs-CRP, thyroid-stimulating hormone, or urinary albumin excretion rate. In Cox analyses, older age and dyslipidemia were the strongest predictors of stroke (*p* < 0.001). Arterial hypertension, diabetes mellitus, and higher NT-pro-BNP levels were also associated with increased stroke risk (*p* < 0.05), whereas CHD anatomical complexity, NYHA functional class, and cyanosis were not. **Conclusions**: In adults with CHD, ischemic stroke was mainly associated with traditional cardiovascular risk factors and elevated NT-pro-BNP levels rather than anatomical disease complexity or functional status.

## 1. Introduction

Congenital heart disease (CHD) affects approximately 1% of live births and represents the most common birth defect worldwide [1,2]. Advances in surgical techniques and medical management have resulted in more than 90% of affected children now surviving into adulthood, with adults currently comprising approximately two-thirds of the entire CHD population [1,3,4]. This demographic shift has transformed CHD from a predominantly pediatric condition into a chronic lifelong disease, creating a growing population facing unique cardiovascular challenges beyond their structural cardiac abnormalities.

Adults with CHD experience a substantially elevated risk of cerebrovascular events compared to the general population. Stroke incidence is 9 to 12 times higher in adults with CHD below age 55 years and 2 to 4 times higher in those aged 55 to 64 years [5,6]. This heightened cerebrovascular risk stems from multiple pathophysiological mechanisms, including chronic hemodynamic stress, paradoxical embolization through residual shunts, atrial arrhythmias, ventricular dysfunction, and systemic inflammation [5,7].

Emerging biomarkers offer promising avenues for enhanced cerebrovascular risk assessment in adults with CHD. N-terminal pro-B-type natriuretic peptide (NT-pro-BNP) demonstrates disease-specific correlations with cardiac dysfunction across various CHD subtypes. In fact, it has been shown that NT-pro-BNP provides prognostic information beyond conventional risk markers and can reliably identify patients at highest risk of cardiovascular events [8,9]. Meanwhile, microalbuminuria, present in approximately one in six adults with CHD, has emerged as an independent predictor of mortality and cardiovascular hospitalization [10]. Importantly, the prognostic value of albuminuria in CHD appears independent of renal function, disease complexity, and functional status [11]. Finally, high-sensitivity C-reactive protein (hs-CRP) reflects the chronic inflammatory state characteristic of CHD and has demonstrated robust associations with adverse outcomes [12,13].

Despite the established prognostic significance of these biomarkers individually, their combined utility for cerebrovascular risk stratification in adults with CHD remains unexplored.

This study aimed to characterize the distribution of microalbuminuria, NT-pro-BNP, and hs-CRP in adults with CHD, to examine their associations with clinical and demographic characteristics, and to evaluate their predictive value for cerebrovascular events, particularly ischemic stroke.

## 2. Methods

### 2.1. Study Design and Participants

This prospective cohort study with longitudinal follow-up enrolled consecutive adults with CHD attending our tertiary referral center from January 2017 through January 2022. Eligible participants were aged 18 years or older with documented structural CHD confirmed by echocardiography, cardiac magnetic resonance imaging, or cardiac catheterization, who underwent routine blood tests and 24-h urine collection during outpatient visits. Patients were excluded if they had undergone cardiac surgery or percutaneous intervention within six months prior to enrollment, had active malignancy, or had estimated life expectancy less than one year. The study protocol received approval from the institutional ethics committee and adhered to the Declaration of Helsinki principles. All participants provided written informed consent prior to enrollment, including consent for research blood and urine sample collection.

### 2.2. Clinical Assessment

Demographic characteristics, cardiovascular risk factors, and medication use were systematically recorded for all participants. Arterial hypertension was defined as office blood pressure ≥ 140/90 mmHg or ongoing antihypertensive treatment. Diabetes mellitus required fasting glucose > 126 mg/dL or use of oral antidiabetics/insulin. Dyslipidemia was identified by Low-Density Lipoprotein (LDL) cholesterol > 130 mg/dL or statin therapy. Patients were classified as current smokers if actively smoking at enrollment [14]. Body mass index (BMI) was calculated as weight in kilograms divided by height in meters squared.

Functional status was classified according to the New York Heart Association (NYHA) functional classification, which categorizes patients based on the degree of symptoms during physical activity. Class I indicates no limitation of physical activity; Class II, slight limitation with symptoms during ordinary activity; Class III, marked limitation with symptoms during less-than-ordinary activity; and Class IV, symptoms present at rest or with minimal physical activity. Systemic ventricular systolic dysfunction was defined according to standard echocardiographic criteria and categorized as moderate to severe dysfunction when the systemic ventricular ejection fraction was <45% for morphologic left ventricles or when qualitative assessment showed moderate or severe systolic impairment in morphologic right systemic ventricles, following established adult CHD echocardiographic practice [15]. Pulmonary hypertension was defined according to the 2022 ESC/ERS guidelines as a high echocardiographic probability (peak tricuspid regurgitation velocity > 3.4 m/s or 2.9–3.4 m/s with additional echocardiographic signs) or, when available, confirmed by right heart catheterization with mean pulmonary artery pressure > 20 mmHg [16]. Medication history encompassed statins, beta-blockers, loop diuretics, antiplatelet agents, oral anticoagulation (vitamin K antagonists or direct oral anticoagulants), angiotensin-converting enzyme inhibitors, angiotensin receptor blockers, calcium channel blockers, and oral iron therapy. Atrial fibrillation and auricular flutter were identified by electrocardiographic documentation. Mechanical valve prostheses were confirmed through medical record review. Systemic ventricular function was assessed by transthoracic echocardiography during routine clinical evaluation. The presence of Down syndrome was determined through review of medical records and confirmed by a previous clinical and/or genetic diagnosis of trisomy 21 documented during prior pediatric or genetic evaluation. Cyanosis was defined as resting oxygen saturation below 90% on pulse oximetry. Cardiac lesions were categorized according to anatomical complexity (simple, moderate, and great) following the 2018 AHA/ACC Guidelines for the Management of Adults with Congenital Heart Disease [17].

### 2.3. Laboratory Measurements

Following a minimum 10-h overnight fast, venous blood samples were obtained for measurement of serum glucose, creatinine, and lipid parameters (total cholesterol, LDL-cholesterol, high-density lipoprotein (HDL) cholesterol, triglycerides and lipoprotein(a)) using spectrophotometry on the Olympus AU 2700 analyzer (Olympus Diagnostic, Hamburg, Germany). NT-pro-BNP was measured by electrochemiluminescence immunoassay using the Siemens Stratus CS Acute Care Diagnostic System (Siemens Healthcare Diagnostics, Inc., Newark, DE, USA) and expressed in pg/mL (picograms per milliliter). Hs-CRP was quantified by immunoturbidimetric assay on the Olympus AU 2700 biochemistry analyzer, with an analytical measuring range of 0.05–20 mg/dL.

Microalbuminuria assessment was performed using 24-h urine collection obtained on the same day as blood sampling. Participants maintained their usual diet but were instructed to abstain from alcohol consumption and strenuous physical activity during the collection period. Urinary albumin was measured in 24-h urine samples using spectrophotometry on the Olympus AU 2700 analyzer. The urinary albumin excretion rate was calculated in micrograms per minute (µg/min) based on the total volume collected and the duration of the collection. All assays were performed following standard laboratory quality control procedures, including calibration of the analyzer and use of internal controls.

### 2.4. Follow-Up and Outcome

Patients were followed prospectively from enrollment until the occurrence of the primary endpoint or end of the study period. Follow-up data were obtained through systematic medical record review and structured telephone interviews. The primary endpoint was ischemic stroke, defined according to the 2017 ACC/AHA Standardized Data Definitions as an acute episode of focal cerebral, spinal, or retinal dysfunction caused by central nervous system infarction, with evidence of acute infarction on neuroimaging or persistence of symptoms for ≥24 h [18].

### 2.5. Statistical Analysis

Continuous variables were summarized using measures of central tendency according to their distribution. Normally distributed variables were expressed as mean ± standard deviation, whereas non-normally distributed variables were presented as median and interquartile range. Group comparisons for continuous data were conducted using either Student’s *t* test for parametric variables or the Wilcoxon rank-sum test for non-parametric variables, as appropriate. Categorical variables were compared using Pearson’s chi-square test.

Time-to-event analyses were performed using univariable Cox proportional hazards regression to evaluate associations between baseline variables and the risk of stroke during follow-up. Given the limited number of stroke events, the analyses were intentionally restricted to univariable models to reduce the risk of model overfitting. Hazard ratios (HRs) were calculated with corresponding 95% confidence intervals (CIs). Analyses were conducted using complete case data given the minimal proportion of missing values. All statistical tests were two-sided, and a *p* value < 0.05 was considered statistically significant. Data analysis was conducted using the Statistical Package for the Social Sciences (SPSS), version 24 (IBM Corp., Chicago, IL, USA).

## 3. Results

### 3.1. Study Population and Stroke Prevalence

Among 372 adults with CHD [median age 34 (23–42) years and 215 (57.8%) men], anatomical complexity was distributed as simple (170 [45.7%]), moderate (121 [32.5%]), and great (81 [21.8%]). Predominant lesions included ventricular septal defect (66 [17.7%]), atrial septal defect (43 [11.6%]), aortic coarctation (36 [9.7%]), and repaired tetralogy of Fallot (32 [8.6%]), while rarer great complexity defects such as dextro-transposition of the great arteries (D-TGA) (21 [5.6%]) and single ventricle (8 [2.2%]) were also represented (Table 1). The category “other lesions” included less frequent conditions followed in our adult CHD clinic (e.g., bicuspid aortic valve, patent foramen ovale, and Marfan syndrome), which were grouped together due to their low individual prevalence.

### 3.2. Demographic and Clinical Characteristics

Baseline characteristics among 372 CHD patients [359 without vs. 13 (3.5%) with stroke] can be seen in Table 2. Patients with stroke were markedly older [median 51 (46–64) vs. 30 (23–40) years; *p* = 0.001], with higher rates of arterial hypertension (30.8% vs. 1.1%; *p* = 0.031), diabetes (23.1% vs. 4.2%; *p* = 0.002), dyslipidemia (61.5% vs. 15.0%; *p* < 0.001), cyanosis (38.5% vs. 10.0%; *p* = 0.001), and treatments including antiplatelets (30.8% vs. 7.5%; *p* = 0.003), oral anticoagulation (53.8% vs. 15.3%; *p* = 0.004), statins (61.5% vs. 6.7%; *p* < 0.001), and oral iron (46.2% vs. 3.6%; *p* < 0.001). On the contrary, no significant differences emerged in sex (53.8% vs. 57.9% men), BMI, CHD complexity distribution, NYHA class ≥II, smoking, renal function, lipid levels, or arrhythmias.

### 3.3. Laboratory and Biomarker Profiles

Table 2 (blood test) revealed significantly higher glucose (104 vs. 94 mg/dL; *p* < 0.001) and NT-pro-BNP levels (369 vs. 64 µg/mL; *p* < 0.001) in stroke vs non-stroke CHD patients. TSH trended higher (3.7 vs. 2.3 mU/L; *p* = 0.084). Meanwhile, no significant differences emerged for renal function, hemoglobin, lipid profile (total/LDL/HDL cholesterol, lipoprotein(a)), hs-CRP, or urine albumin excretion rate (all *p* > 0.30).

### 3.4. Univariable Predictors of Stroke

Univariable Cox proportional hazards analyses were performed to assess the association between baseline variables and the risk of stroke over time. Increasing age and dyslipidemia showed the strongest associations with stroke risk (*p* < 0.001), while arterial hypertension and diabetes mellitus were also related to a higher likelihood of stroke (*p* < 0.05). Higher NT-pro-BNP levels were modestly but significantly associated with stroke occurrence (*p* = 0.033). In contrast, CHD anatomical complexity and NYHA functional class were not associated with stroke risk, and cyanosis showed only a non-significant trend toward increased risk. Detailed results of the univariable Cox analyses are presented in Table 3.

## 4. Discussion

This prospective cohort study evaluated the potential role of circulating biomarkers for cerebrovascular risk stratification in adults with CHD. In exploratory analyses, dyslipidemia and higher NT-pro-BNP levels were associated with an increased risk of ischemic stroke, highlighting the potential interaction between acquired cardiovascular risk factors and underlying hemodynamic stress in aging CHD survivors.

### 4.1. Prevalence

Stroke prevalence in our adult CHD cohort was 3.5%, at the lower end of the range described in previous series, which reported cumulative stroke rates of 3.1–7.7% by the time patients reached 64 years of age [6,19,20,21]. This finding should be interpreted considering the younger age of our population and the fact that patients were followed at a tertiary referral center, where structured surveillance and optimized medical management are routinely implemented. These factors may have contributed to a lower observed stroke prevalence compared with less intensively managed cohorts.

### 4.2. Cardiorenal Biomarkers

Higher NT-pro-BNP levels were associated with stroke occurrence in our cohort. NT-pro-BNP reflects myocardial stress and neurohormonal activation—such as ventricular strain, atrial stretch, and shunt-related volume overload—which may increase embolic risk in patients with CHD [8]. Previous multicenter studies have demonstrated that NT-pro-BNP provides prognostic information for adverse cardiovascular outcomes in adults with CHD by integrating multiple pathophysiological processes into a single measurable marker [8].

In our study, patients with higher NT-pro-BNP concentrations showed a greater likelihood of stroke events. Elevated NT-pro-BNP levels may reflect subclinical cardiac dysfunction, atrial arrhythmia susceptibility, or abnormal hemodynamics, all of which are recognized contributors to cardioembolic stroke. Therefore, NT-pro-BNP may represent a useful biomarker to help identify patients with increased cerebrovascular vulnerability within the adult CHD population.

The observed association between dyslipidemia and stroke likely reflects the growing influence of acquired cardiovascular risk factors in the aging CHD population [3]. Although embolic mechanisms remain the predominant cause of stroke in adults with CHD, advancing age is associated with an increasing overlap with traditional atherosclerotic risk profiles, in which dyslipidemia may contribute to cerebrovascular risk [22,23]. However, the absence of a clear relationship between individual lipid levels and stroke events in our cohort may partly reflect the widespread use of statin therapy and partial lipid normalization.

In contrast, hs-CRP and microalbuminuria did not show significant associations with stroke in our cohort. Although both biomarkers have been linked to adverse cardiovascular outcomes in adults with CHD—including heart failure, hospitalization, and mortality—their contribution to cerebrovascular risk may be less pronounced in this population, where embolic mechanisms are thought to predominate [11,12,13,24,25]. In addition, these biomarkers primarily reflect systemic inflammation and endothelial or renal dysfunction rather than the hemodynamic abnormalities and arrhythmia-related mechanisms that more directly contribute to cardioembolic stroke in CHD. Consequently, their prognostic value may be greater for overall cardiovascular outcomes than for the specific prediction of cerebrovascular events.

### 4.3. Clinical CHD Markers and Stroke Risk

Several factors may explain why CHD anatomical complexity, NYHA functional class, and cyanosis were not significantly associated with stroke in our cohort. First, the relatively small number of stroke events limited statistical power and may have reduced the ability to detect associations with disease-specific characteristics. In addition, our cohort consisted of a well-followed tertiary-care adult CHD population receiving contemporary medical management, including frequent use of antiplatelet and anticoagulant therapies. Such preventive strategies may attenuate the thromboembolic risk traditionally associated with more complex lesions or advanced functional impairment.

Another explanation is that circulating biomarkers such as NT-pro-BNP may capture subclinical hemodynamic stress or early cardiac dysfunction that is not fully reflected by anatomical complexity or NYHA class. Elevated NT-pro-BNP levels may therefore identify patients with occult atrial or ventricular strain who appear clinically stable but remain at increased risk of cardioembolic events. Finally, cyanosis in CHD often reflects underlying anatomical or physiological conditions—such as residual shunts or complex circulatory pathways—rather than representing a direct marker of thromboembolic risk. Although chronic hypoxemia has been associated with secondary erythrocytosis, hyperviscosity, and paradoxical embolism [26], these mechanisms alone do not appear to be the main determinants of stroke risk. Instead, other clinical factors, including atrial arrhythmias, ventricular dysfunction, and Fontan circulation, seem to be stronger predictors of thromboembolic events [27].

Taken together, these findings suggest that circulating biomarkers reflecting hemodynamic stress or acquired cardiovascular risk may provide additional information for stroke risk stratification in adults with CHD. However, these observations should be interpreted cautiously given the limited number of outcome events.

### 4.4. Limitations of the Study

Our findings should be interpreted in light of several limitations. First, the relatively small number of stroke events (*n* = 13) may have limited statistical power; therefore, the findings should be considered exploratory and hypothesis-generating. Second, the single-center design at a tertiary referral institution may limit the generalizability of the findings, particularly given the heterogeneous nature of the adult CHD population included in this study, which ranged from simple to complex lesions with diverse hemodynamic profiles, arrhythmia burdens, and embolic risks. Third, residual confounding from unmeasured factors—such as transcranial Doppler assessment for right-to-left shunting—cannot be excluded. Fourth, indication bias may have influenced our results, as antithrombotic therapy was selectively prescribed to patients perceived to be at higher risk. Furthermore, dyslipidemia was defined using LDL cholesterol levels or statin therapy, which may introduce some overlap between exposure definition and treatment status. Finally, biomarker levels were assessed at a single time point, and longitudinal measurements might have provided additional prognostic information.

Despite these limitations, the study has notable strengths, including its prospective design, standardized biomarker assessment, and long-term follow-up in a well-characterized adult CHD cohort. To our knowledge, this is one of the first prospective studies to systematically evaluate cardiorenal biomarkers—including NT-pro-BNP, hs-CRP, and microalbuminuria—in relation to ischemic stroke risk in adults with CHD.

## 5. Conclusions

In this prospective cohort of adults with CHD, dyslipidemia and higher NT-pro-BNP levels were associated with an increased risk of ischemic stroke in exploratory analyses. These findings suggest that circulating biomarkers reflecting cardiovascular stress and acquired risk factors may provide additional information for cerebrovascular risk assessment in adult CHD. However, given the limited number of events, the results should be interpreted cautiously and require confirmation in larger multicenter studies.

## Figures and Tables

**Table 1:** Types of CHD according to their anatomical complexity.

Type of Congenital Heart Disease	Simple	Moderate	Great	Total
Ventricular septal defect (VSD)	60	0	6	66
Atrial septal defect (ASD)	38	0	5	43
Aortic coarctation	0	36	0	36
Tetralogy of Fallot (repaired)	0	32	0	32
Pulmonary stenosis	30	0	1	31
Dextro-transposition of the great arteries (D-TGA)	0	0	21	21
Partial atrioventricular septal defect (pAVSD)	0	16	2	18
Complete atrioventricular septal defect (cAVSD)	0	13	4	17
Congenitally corrected TGA (ccTGA)	0	0	12	12
Single ventricle	0	0	8	8
Double outlet right ventricle (DORV)	0	0	8	8
Patent ductus arteriosus (PDA)	8	0	0	8
Pulmonary atresia	0	0	7	7
Aortic stenosis	5	0	0	5
Ebstein anomaly of the tricuspid valve	0	5	0	5
Tricuspid atresia	0	0	3	3
Subvalvular/supravalvular pulmonary stenosis	0	3	0	3
Subvalvular/supravalvular aortic stenosis	0	3	0	3
Anomalous pulmonary venous drainage	0	2	1	3
Truncus arteriosus	0	0	2	2
Aorto-venous fistula (systemic or pulmonary)	1	0	1	2
Systemic venous anomaly	1	0	0	1
Other lesions (bicuspid aortic valve, mitral/aortic insufficiency, valvular prolapse, subaortic membrane, patent foramen ovale, Marfan syndrome)	23	11	0	34
Total	170	121	81	372

CHD: congenital heart disease.

**Table 2:** Demographic, clinical and blood test data in CHD patients according to stroke.

	CHD Patients		*p* *
	Without Stroke	With Stroke	
CHD patients, *n*	359	13	
Age, years	30 (23–40)	51 (46–64)	<0.001
Sex (male), *n*	208 (57.9%)	7 (53.8%)	0.769
BMI, kg/m^2^	23 (21-28)	26 (22–27)	0.135
Great CHD complexity, *n*			0.716
Mild	165 (46.0%)	5 (38.5%)	
Moderate	117 (32.6%)	4 (30.8%)	
Great	77 (21.4%)	4 (30.8%)	
NYHA functional class (≥2), *n*	20 (5.6%)	1 (7.7%)	0.854
Arterial hypertension, *n*	4 (1.1%)	4 (30.8%)	0.031
Diabetes mellitus, *n*	15 (4.2%)	3 (23.1%)	0.002
Dyslipidemia, *n*	54 (15.0%)	8 (61.5%)	<0.001
Smoker, *n*	20 (5.6%)	1 (7.7%)	0.272
Blood test			
Glucose, mg/dL	94 (88–101)	104 (97–111)	<0.001
Creatinine, mg/dL	0.9 (0.8–1.0)	0.9 (0.8–1.1)	0.505
GFR, mL/min/1.73 m^2^	90 (79–104)	85 (70–104)	0.325
Hemoglobin, mg/dL	15 (14–16)	14 (14–16)	0.881
Total cholesterol, mg/dL	159 (136–186)	162 (135–184)	0.845
LDL cholesterol, mg/dL	92 (71–114)	91 (78–110)	0.828
HDL cholesterol, mg/dL	49 (41–56)	50 (41–56)	0.988
Lipoprotein(a), mg/dL	12 (5–36)	15 (4–49)	0.710
Ferritin, ng/mL	36 (18–69)	25 (15–44)	0.314
NT-pro-BNP, pg/mL	64 (21–172)	369 (218–604)	<0.001
Hs-CRP, mg/dL	0.15 (0.1–0.5)	0.2 (0–150)	0.557
TSH, mU/L	2.3 (1.5–3.4)	3.7 (1.6–6.2)	0.084
Rate of urine albumin excretion, µg/min	4.9 (0–11)	12.1 (0–150)	0.557
Treatment			
Antiplatelet, *n*	27 (7.5%)	4 (30.8%)	0.003
Oral anticoagulation, *n*	55 (15.3%)	7 (53.8%)	0.004
Beta-blockers, *n*	47 (13.1%)	5 (38.5%)	0.011
ACE inhibitors, *n*	35 (9.7%)	3 (23.1%)	0.132
ARBs, *n*	17 (4.7%)	1 (7.7%)	0.640
Calcium channel blockers, *n*	13 (3.6%)	1 (7.7%)	0.466
Loop diuretics, *n*	51 (14.2%)	6 (46.2%)	0.002
Statins, *n*	24 (6.7%)	8 (61.5%)	<0.001
Oral iron, *n*	13 (3.6%)	6 (46.2%)	<0.001
Mechanical valve prosthesis, *n*	18 (5.0%)	4 (30.8%)	0.324
Systemic ventricular dysfunction ^#^, *n*	57 (15.9%)	2 (15.4%)	0.945
Cyanosis, *n*	36 (10.0%)	5 (38.5%)	0.001
Arterial pulmonary hypertension, *n*	30 (8.4%)	1 (7.7%)	0.875
Down syndrome, *n*	34 (9.5%)	0 (0.0%)	0.997
Atrial fibrillation & auricular flutter	22 (6.1%)	2 (15.4%)	0.242

CHD: congenital heart disease, *n*: number of patients, BMI: body mass index, NYHA: New York Heart Association, GFR: glomerular filtration rate, LDL: Low-Density Lipoprotein, HDL: High-Density Lipoprotein, NT-pro-BNP: NT-pro-brain natriuretic peptide, Hs-CRP: high sensitivity C reactive protein, TSH: thyroid-stimulating hormone; ACE: angiotensin converting enzyme, ARBs: angiotensin receptor blockers, ^#^ moderate to severe systemic ventricular dysfunction. The data are expressed as median and (25–75) percentiles and as number and percentage. * Continuous data without normal distribution by Mann–Whitney test.

**Table 3:** Univariable Cox proportional hazards analysis of baseline variables associated with stroke in patients with CHD.

	Univariate Analysis	
	HR (95% CI)	*p*
Age, years	1.06 (1.03–1.09)	<0.001
CHD complexity ^a^	1.02 (0.51–2.02)	0.973
NYHA class ^b^	0.88 (0.11–6.78)	0.900
Arterial hypertension, yes	3.28 (1.01–10.73)	0.049
Diabetes mellitus, yes	4.50 (1.23–16.42)	0.023
Dislipidemia, yes	10.06 (3.02–33.47)	<0.001
NT-pro-BNP, pg/mL	1.001 (1.000–1.001)	0.033
Cyanosis, yes	2.94 (0.91–9.49)	0.071

CHD: congenital heart disease, NYHA: New York Heart Association functional class, NT-pro-BNP: NT-pro-brain natriuretic peptide, HR: Hazard ratio, CI: confidence interval. ^a^ CHD mild and moderate vs. great complexity, ^b^ NYHA class I vs. patients with class ≥ 2.

## Data Availability

The data supporting the findings of this study are not publicly available due to the absence of participant consent for data sharing and the sensitive nature of the research.
